# Using the method of judgement analysis to address variations in diagnostic decision making

**DOI:** 10.1186/1756-0500-5-139

**Published:** 2012-03-13

**Authors:** Helen C Hancock, James M Mason, Jerry J Murphy

**Affiliations:** 1 School of Medicine and Health, Durham University, Queen's Campus, Wolfson Research Institute, University Boulevard, Stockton-on-Tees, UK; 2 Darlington Memorial Hospital, County Durham & Darlington NHS Foundation Trust, Hollyhurst Road, Darlington, UK

## Abstract

**Background:**

Heart failure is not a clear-cut diagnosis but a complex clinical syndrome with consequent diagnostic uncertainty. Judgment analysis is a method to help clinical teams to understand how they make complex decisions. The method of judgment analysis was used to determine the factors that influence clinicians' diagnostic decisions about heart failure.

**Methods:**

Three consultants, three middle grade doctors, and two junior doctors each evaluated 45 patient scenarios. The main outcomes were: clinicians' decisions whether or not to make a diagnosis of suspected heart failure; the relative importance of key factors within and between clinician groups in making these decisions, and the acceptability of the scenarios.

**Results:**

The method was able to discriminate between important and unimportant factors in clinicians' diagnostic decisions. Junior and consultant physicians tended to use patient information similarly, although junior doctors placed particular weight on the chest X-Ray. Middle-grade doctors tended to use information differently but their diagnostic decisions agreed with consultants more frequently (k = 0.47) than junior doctors and consultants (k = 0.23), or middle grade and junior grade doctors (k = 0.10).

**Conclusions:**

Judgment analysis is a potentially valuable method to assess influences upon diagnostic decisions, helping clinicians to manage the quality assurance process through evaluation of care and continuing professional development.

## Background

Heart failure is not a disease per se, but a complex clinical syndrome [1]. Unlike kidney or lung failure there is no easily measured organ function parameter to determine a diagnosis. Diagnosis and management are based on clinicians' decisions, informed by a number of factors including the patient's medical history, physical examination, electrocardiogram and chest x-ray [2]. With early accurate diagnosis and appropriate treatment prognosis can be substantially improved. However, there is evidence to suggest that diagnosis is missed in up to half of cases [3-5]. Diagnosis and management are complex; symptoms are often non-specific and physical signs are difficult to elicit. An echocardiogram, measuring left ventricular ejection fraction, is used to classify common forms of heart dysfunction although various methods of measurement give different results, and there is no definitive result below which heart failure can be confirmed [6]. Managing uncertainty is central to clinical practice and requires the linking of experience and evidence: this places specialists at an advantage, but junior doctors, nurses and general practitioners often make diagnostic decisions about heart failure.

National Service Frameworks (NSFs) and clinical guidelines are fundamental to clinical governance programmes, aiming to improve standards and reduce unacceptable variations in clinical practice [7]. However, neither can be easily applied to complex decision-making and their recommendations need contextualising to be relevant: no process exists to ensure this routinely occurs and variation in care remains common. The lack of information about the sources of variability in diagnosis and care means that variability may be simplistically viewed as inappropriate, but may appropriately reflect individual patient needs, activity recording variations or alternative but valid traditions of care.

Existing methods of assessing clinicians' decision-making tend to polarise around the process of decision-making (e.g. sociological enquiry) or on its outcome (e.g. audit). Clinical examinations or Objective Structured Clinical Examinations (OSCEs) are popular [8], but are removed from real life situations [9]. Observations of clinicians in practice aim to address these concerns [10], but the focus is on the decision rather than on the information that informs it. Similarly, 360° appraisals are used to assess performance [11] although they also focus on decisions made and are vulnerable to poorly informed or biased reports from colleagues [12]. Clinical decisions often involve evaluating multiple cues in situations of uncertainty, thus, to understand and address unacceptable variations in care, assessment should link the process of decision-making (i.e. the factors that inform a decision) and the decision made.

Judgement analysis links the process (i.e. how clinicians use information to reach a decision) and outcome of clinical decisions [13,14], The method involves identifying information used in a diagnostic or treatment context. This information is expressed as a number of variables or factors used within a series of scenarios. Judgement analysis offers a means of examining the information used by clinicians in their decision making: it can be used to compare a decision to an agreed standard, such as a protocol or guideline, or to that of another clinician or reference group. Results can be used to direct education and training and to help determine practical limitations of guidelines. In essence, judgment analysis provides a systematic variation of the factors that affect decisions and, thus, provides insight about how doctors use information in their decision making, and how and why they vary. Inappropriate variation in health care is of primary concern but is very poorly researched. While it might seems obvious that, in general, senior clinicians might be better diagnosticians than their junior colleagues, seniority is not a marker of accuracy per se. Furthermore it is not obvious how senior clinicians use information to make better decisions; this information could be used to guide education and training. The method of judgment analysis offers an important way of addressing variation in decision making, but is relatively unused. This paper describes pilot work necessary to develop its use and will be of help to other research teams considering using the technique.

### Applying judgment analysis to diagnostic decisions about heart failure

The example used in this paper demonstrates how judgement analysis can be used to assess clinicians' diagnostic decisions about heart failure:

(i) quantifying how a range of factors inform clinicians' diagnostic decisions about heart failure

(ii) measuring any differences in these factors within and between groups of consultant, middle grade and junior doctors

(iii) assessing the acceptability and feasibility of using the scenarios.

## Methods

Ethical guidance was obtained from County Durham and Tees Valley 1 Research Ethics Committee. Given the nature of the study, ethical approval was deemed by the Chair of this committee not to be required. The research was partially funded by an academic grant from Darlington Memorial Hospital. Study conduct conformed to the principles of The Declaration of Helsinki.

### Phase 1. Developing clinical scenarios

Clinical variables affecting diagnosis were identified by a consensus panel of 6 cardiology consultants who identified eight factors as key variables in the diagnosis of heart failure; these are listed in Table 1. For each variable, levels of normality (e.g. normal and abnormal (low or high)) were also agreed (see Table 1). These variables were used to generate a number of clinical scenarios; a key decision is whether scenarios should present information as found in every day clinical practice or whether to use more artificial, non-correlated, orthogonally-based scenarios [15]. The latter may be better at determining the importance of variables affecting decisions but may lose some clinical relevance. Applying the principle of representativeness [16] (i.e. typical of real world scenarios), variables and their levels were entered into SPSS (version 14) and scenarios were generated using an orthogonal design, random number generator. From this, 81 different scenarios were produced; these were transposed into clinical scenarios.

**Table 1:** The Eight Key Variables Included in the Scenarios and their Levels

Variable	Levels 1	2	3
1. Orthopnoea	No	Yes	

2. Pitting oedema	None	Mild, around ankles	Marked, above knees

3. Ischaemic heart disease	No	Yes	

4. Heart rate	60-100	< 60	> 100

5. Lung signs	No crackles	Crackles	

6. Chest x-ray (lungs)	Normal	Upper lobe veins enlarged	Alveolar oedema

7. Heart size	Normal	Enlarged	

8. Electrocardiogram	Normal	Bundle Branch Block	"Ischaemic"

Key: Scale 1-3 where 1 = normal, and 2 and 3 represent increasing abnormality

In order that as much ecological validity as possible was retained, the variables were presented so that the scenarios reflected the format found in practice. Thus, while, for example, a heart rate of 60-100 beats per minute (bpm) was considered normal, < 60 bpm low and > 100 bpm high, a scenario which included 'heart rate: high' might read HR 126 bpm. The following is an example:

*A 74-year-old patient presents to A&E with breathlessness. She has no history of IHD. She can lay flat without increased breathlessness. She has no ankle oedema. Her heart rate is 120 bpm; ECG shows T wave changes suggestive of ischaemia. There are crackles at both lung bases; CXR shows pulmonary oedema; heart size is normal. This patient has suspected heart failure: Yes/No (circle one)*.

The 81 scenarios were discussed with a Consultant Cardiologist to ensure that the format reflected real scenarios and which, if any, should be excluded. 41 scenarios were excluded on the basis that they were not plausible, or that they represented a healthy person. Five scenarios were duplicated in order to determine the consistency of individual responses; these repeated scenarios, called 'hold out' cases, were not included in the analysis. Thus, a total of 45 scenarios were included.

### Phase 2. Completion of scenarios by participants

Three cardiology consultants, three middle grade doctors (two Registrars and one Senior House Officer), and two junior doctors (Foundation year 1) were sent the same 45 scenarios in the same order in one document, they were asked to note the time it took to complete the scenarios and evaluate the format. Clinicians were encouraged to make decisions as they would in practice. For each scenario they were asked whether or not they would make a diagnosis of suspected heart failure (where heart failure becomes the 'working diagnosis').

In addition to the scenarios, the doctors were asked to complete a number of other questions. These were: (i) participant demographics; (ii) a list of factors to be rated by participants according to their importance in establishing a diagnosis of heart failure, where 1 = not important, 2 = slightly (or occasionally) important, 3 = moderately (or often) important, and 4 = very (or always) important and the variables were: gender, age, orthopnoea, pitting oedema, history of IHD, heart rate, lung signs, CXR (lungs, heart size, ECG, JVP, Gallop rhythm, out of hours consultation (6 pm-6 am), others (specify); (iii) feedback about the scenarios including: how long it took to complete the scenarios; to what extent the scenarios adequately reflected the information used when making diagnoses with real patient [exactly, very well, quite well, not very well or not at all]; how the scenarios could be improved; any other information that would have been useful in the scenarios; whether or not participants would have preferred to have received the scenarios by email; final comments and suggestions.

### Phase 3. Data analysis

The dependent variable within the analyses was the decision whether or not to make a diagnosis of suspected heart failure. Data analysis used the Conjoint procedure within SPSS [17], which generated a relative utility (importance) score for each variable and variable level [17]. As utility scores share a common metric, it is possible to calculate the relative influence of each variable. Utility scores vary between -1 and 1 with zero denoting no influence and larger magnitudes corresponding to a greater negative or positive contribution. Repeated scenarios were not included in the Conjoint analysis.

### Statistical comparisons

Descriptive and inferential statistical analyses were conducted using SPSS version 14. Decision repeatability and consistency within and between clinician groups were analysed using categorical agreement analysis (kappa). The kappa score indicates the level of agreement between or within raters on a scale from zero to one, where the level of agreement is poor (< 0.2), fair (≥ 0.2 to < 0.4), moderate (≥ 0.4 to < 0.6), good (≥ 0.6 to < 0.8) or very good (≥ 0.8).

## Results

### Clinicians' decisions about whether or not to make a diagnosis of suspected heart failure

The repeatability of diagnostic decisions within clinician groups for the five repeat scenarios was between moderate and very good (Table 2). The consistency of diagnostic decision-making across the 40 scenarios was variable both within and between clinical groups, never exceeding moderate agreement. Agreement was higher between consultants and middle grade doctors (k = 0.47) than between consultants and junior grade doctors (k = 0.23) or middle grade and junior grade doctors (k = 0.10), suggesting that there were important diagnostic variations that should be explored within the clinical team as a quality assurance issue.

**Table 2:** Agreement Scores (Kappa) for Repeatability and Consistency of Diagnoses of Suspected Heart Failure

		Repeatability^+^		Consistency*	
**Seniority**	**N**	**Within group**	**Consultant**	**Middle Grade**	**Junior**

Consultant	3	0.53	0.25		

Middle Grade	3	0.73	0.47	0.16	

Junior	2	1	0.23	0.10	0.47

+ Repeatability of categorical diagnostic decisions (yes/no) for 5 repeated scenarios using Kappa

* Within Group: consistency of categorical diagnostic decisions (yes/no) for 40 scenarios within each level of seniority using Kappa

Between Groups: consistency of categorical diagnostic decisions (ordinal levels) for 40 scenarios comparing different level of seniority using weighted Kappa

### The relative importance of the variables within and between clinician groups

**Figure 1:**
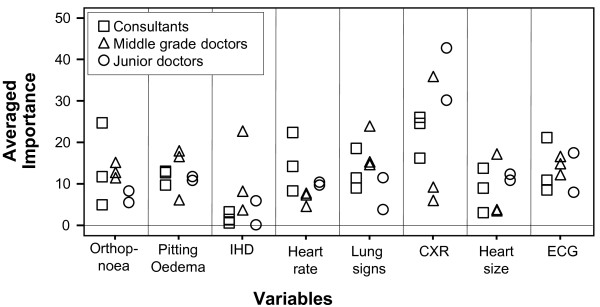
**The Importance of Variables: Comparison Between and Within Groups**.

The average importance of the variables influencing decisions for each group is presented in Table 3 permitting visual comparisons between groups. Importance scores sum to 100 showing the relative importance of each variable for each group. As a demonstration project, no formal statistical comparisons were planned but an interesting trend was apparent. Junior doctors' use of information reflected more closely that of the consultants while middle grade doctors often differed substantially. The two most important variables for consultants were chest x-ray and heart rate; and, for middle grade doctors lung signs and ischaemic heart disease. These two professional groups used a broader range of information, whereas junior doctors focused on chest x-ray findings. Average utility scores by group provide valuable information about the use of information but conceals variability within groups. Despite all clinicians receiving the same scenarios, there was considerable variation within clinician groups as illustrated in Figure 1.

**Table 3:** The Average Importance of the Variables by Clinician Group

Variable	Consultant Score	Middle Grade Dr Score	Junior Dr Score
Orthopnoea	4.86	12.29	5.39

Pitting Odema	9.69	16.28	11.01

Ischaemic heart disease	3.37	22.41	5.97

Heart rate	22.39	4.36	10.50

Lung signs	9.04	23.66	3.68

Chest x-ray	26.01	5.7	42.92

Heart Size	13.94	3.2	12.4

Electrocardiogram	10.67	11.97	8.08

**Total Score**	100	100	100

### The acceptability of the scenarios

#### (i) Time Taken

Time taken to complete the scenarios varied substantially: a mean of 28 min and range of 10 to 45 min. Consultants ranged from 10 to 25 min and junior doctors from 25 to 45 min. All three middle grade doctors reported taking 30 min.

#### (ii) Overall Format

Consultant cardiologists and middle grade doctors reported that the scenarios reflected clinical situations 'quite well' (n = 5) or 'very well' (n = 1), while junior doctors (n = 2) reported that they did so 'exactly'.

#### (iii) Preference for Email or Hard Copy of Scenarios

Half of all respondents (n = 6) reported a preference for a hard copy rather than electronic copy of the scenarios. There was variability within and between groups.

## Discussion

Clinical judgment analysis links how clinicians make diagnostic decisions with what decisions are made. This study demonstrated considerable variations in heart failure diagnostic decision-making within and between clinical grades. The method was able to discriminate between influential and uninfluential factors diagnostic decisions both within and between clinician groups. The development, completion and analysis of the scenarios was feasible and acceptable to clinicians.

Without a reference standard answer for each scenario it is not possible to assess the accuracy of diagnoses made, or appropriateness of variations. One possible explanation for variability is that senior clinicians possessed experience and extant knowledge to inform their decisions, junior doctors followed protocols and middle grade doctors employed a combination of the two. Clinical guidelines and protocols seek to standardise practice and to eliminate variability in patient care. However, evidence-based guidelines only provide for 'usual' or 'average' clinical scenarios. Some diagnostic variation may have resulted from clinicians' perceptions about individual patient needs within the scenarios. Notwithstanding the different weight placed on the factors informing diagnostic decisions, consultant and middle grade doctor decision-making was similar. However agreement between junior doctors and more senior grades was only poor to fair - a finding that cannot be disregarded as it may reflect real differences in clinical care.

It is unclear why middle grade doctors made markedly different use of diagnostic information. The method's strength is that it unmasks values used in decision-making that may be incorporated into the training context to explore quality-of-care issues.

### Limitations

This demonstration study is based on a small sample and precludes definitive conclusions. Clinicians were asked to complete the scenarios in the same way as they would in practice. While the scenarios were constructed to reflect real clinical situations faced by them, it is not possible to recreate the same pressures as life in clinical practice. Participants' responses to questions about the acceptability of the scenarios supported their current format.

### Relevance to practice

Changing roles within health care, with more junior staff taking on greater responsibility, have been accompanied by increased public and professional scrutiny [18,19]. This has occurred, in part, because patients, society, and the professions need to be assured that individual clinicians are not only qualified, but consistently provide high quality, safe care for patients. All clinical practise involves uncertainty in diagnosis, prognosis and treatment, and adverse health care events causing physical or psychological injury to patients are surprisingly common [20]. In the UK, adverse events take place in about 10% of NHS admissions and cost £2 billion a year; 400 people die or are seriously injured in adverse events; and, over £500 million was paid out in clinical negligence claims in 2004/2005 [21]. Judgement analysis offers a means of quantifying the factors that influence complex decisions made in clinical practice, thus potentially reducing the likelihood of adverse events when linked to continuing professional development.

Clinical judgement analysis has been applied previously in a number of contexts, supporting our findings of substantial clinical variability when decisions involve complexity [15,22-26]. The method needs developing beyond measuring variations to demonstrate improved consistency and quality of care. Our findings are the first part of a research programme to develop a targeted education and training tool to promote quality and safety within clinical teams.

## Conclusions

Variable clinical decision-making has important implications for diagnosis and management. This is particularly important for heart failure in the hospital setting, since junior and middle grade doctors often make diagnostic decisions. While judgement analysis may help explain and quantify diagnostic variation permitting its discussion as a quality issue within the clinical team. Findings may subsequently positively shape future clinical guidelines, in particular identifying areas of variation and contention. Rather than the imposition of an external clinical governance agenda, the use of this method represents an opportunity for clinical teams to lead the quality assurance process and to differentiate between unacceptable and acceptable variability in care. Judgment analysis usefully captures the determinants of clinicians' diagnostic decisions about heart failure. This pilot study demonstrates the potential for the method to facilitate quality assurance within the clinical team by enabling teams to explore variations, reassess educational support, and make appropriate use of (or modify) guidelines. Further adequately-powered research is required to realize this potential and inform clinical management.

## Authors' contributions

The named authors made the following contributions: HH and JM: substantial contributions to the conception and design, analysis and interpretation of data; drafting the article and revising it critically for important intellectual content; and final approval of the version to be published. JM: substantial contributions to study design and the acquisition of data, drafting the article and revising it critically for important intellectual content; drafting the article and revising it critically for important intellectual content; and final approval of the version to be published. All authors read and approved the final manuscript.

## Competing interests

The authors declare that they have no competing interests.
